# Apneic Tracheal Stenting Using Femorofemoral Venoarterial Extracorporeal Membrane Oxygenation in a Coronavirus Disease 2019 (COVID-19) Patient With Critical Airway Obstruction: A Case Report

**DOI:** 10.7759/cureus.99671

**Published:** 2025-12-19

**Authors:** Akira Iwamizu, Shohei Kaneko, Ryosuke Shintani, Haruka Yokoyama, Taiga Ichinomiya, Tetsuya Hara

**Affiliations:** 1 Department of Anesthesiology and Intensive Care Medicine, Nagasaki University Graduate School of Biomedical Sciences, Nagasaki, JPN

**Keywords:** airway management, covid-19, differential hypoxia, infection prevention and control, sars-cov-2, tracheal stent, venoarterial extracorporeal membrane oxygenation (va ecmo)

## Abstract

Palliative tracheal stenting for severe central airway obstruction in patients with coronavirus disease 2019 (COVID-19) presents a dual challenge: the imminent risk of acute asphyxia during anesthetic induction and the risk of viral transmission to healthcare professionals via aerosols. While extracorporeal membrane oxygenation (ECMO) can serve as a safety bridge to facilitate apneic interventions, conventional venovenous ECMO may not be feasible in patients with superior vena cava (SVC) compression. Femorofemoral venoarterial ECMO (VA-ECMO) is an alternative; however, it carries a substantial risk of differential hypoxia, particularly in patients with poor native lung function. We report a case of a 41-year-old woman with COVID-19 pneumonia and critical tracheal stenosis (luminal diameter 2 mm) caused by mediastinal lymphadenopathy. Given the prohibitive risk of airway collapse and the need for aerosol containment, she underwent successful apneic tracheal stent placement under femorofemoral VA-ECMO support. This approach was employed because SVC compression precluded upper body cannulation, and differential hypoxia was managed using a multifaceted physiological strategy without circuit modification.

A combination of high-flow ECMO, deep anesthesia, pharmacologic cardiac output suppression, and anemia correction effectively mitigated the risk of differential hypoxia. This case demonstrates that femorofemoral VA-ECMO is a safe and effective option for facilitating apneic high-risk airway procedures, simultaneously ensuring airway security and infection control when conventional strategies are insufficient. Further case accumulation is needed to determine the validity and generalizability of these findings.

## Introduction

Coronavirus disease 2019 (COVID-19), caused by severe acute respiratory syndrome coronavirus 2 (SARS-CoV-2), presents significant challenges for perioperative airway management due to its high transmissibility via aerosols and droplets [1]. In patients with COVID-19, airway procedures, including intubation, bronchoscopy, and tracheal interventions, require careful planning to minimize aerosol generation and protect healthcare professionals. Tracheal stent placement is commonly indicated for central airway obstruction caused by malignant tumors, mediastinal lymphadenopathy, or postintubation tracheal stenosis. These procedures typically require general anesthesia with rigid bronchoscopy and positive-pressure ventilation, often involving high-frequency jet ventilation [2]. However, jet ventilation is a high-risk aerosol-generating procedure due to air leakage from the bronchoscope [3], which significantly increases viral transmission risk to healthcare professionals. To reduce aerosol exposure, venovenous extracorporeal membrane oxygenation (VV-ECMO) has been employed to facilitate airway interventions under apnea in select patients with COVID-19 [4].

While VV-ECMO can be established via femorojugular or femorofemoral cannulation, upper body venous access, such as the internal jugular vein or superior vena cava (SVC), is often preferred to minimize recirculation and optimize oxygenation [5]. However, mediastinal masses or lymphadenopathy can severely compress these upper body veins, complicating VV-ECMO implementation [6]. Given the limitations of VV-ECMO in such clinical contexts, venoarterial ECMO (VA-ECMO) offers an alternative for gas exchange and perfusion. Nevertheless, femorofemoral VA-ECMO carries the risk of differential hypoxia, in which well-oxygenated blood from ECMO preferentially perfuses the lower body, potentially causing upper body hypoxemia when native cardiac output is preserved, and pulmonary function is poor [7]. Other strategies to avoid differential hypoxia include left ventricular venting and hybrid ECMO configurations (e.g., venoarteriovenous (V-AV) ECMO) [7]. However, reports of tracheal procedures performed under femorofemoral VA-ECMO without positive pressure ventilation remain limited. Femorofemoral VA-ECMO-assisted tracheal interventions often involve intermittent or backup ventilation [8,9], with few cases of true apneic management [9].

Herein, we report a case of a patient with COVID-19 presenting with severe tracheal compression and SVC stenosis due to metastatic mediastinal lymphadenopathy, who underwent successful tracheal stent placement without mechanical ventilation under femorofemoral VA-ECMO support.

## Case presentation

A 41-year-old woman (height, 165 cm; weight, 47 kg) with a history of ileal adenocarcinoma diagnosed five years earlier had undergone partial ileal resection. She subsequently developed pelvic recurrence and was receiving palliative chemotherapy. The patient presented to a local hospital with fever and cough and was diagnosed with COVID-19, after which she was admitted for further care. Chest computed tomography (CT) at admission to the local hospital revealed marked mediastinal lymphadenopathy causing severe tracheal stenosis. As her dyspnea progressed, she was referred to our institution for tracheal stent placement to relieve symptoms.

On admission, the patient’s vital signs were as follows: body temperature, 36.8°C; blood pressure, 131/98 mmHg; heart rate, 91 beats/minute; and oxygen saturation, 99% on 2 L/minute supplemental oxygen via nasal cannula. However, her dyspnea gradually worsened, necessitating respiratory support with a high-flow nasal cannula (flow rate, 50 L/minute; oxygen concentration, 40%). She developed tachypnea and marked hypoxemia unless positioned in the left decubitus position. Auscultation revealed decreased breath sounds in the right lung field. There were no signs of jugular venous distension or facial swelling. Laboratory tests showed a white blood cell count of 5,500/μL, C-reactive protein level of 0.52 mg/dL, and hemoglobin level of 9.7 g/dL, indicating mild anemia and low-grade inflammation. Nasopharyngeal polymerase chain reaction confirmed SARS-CoV-2 infection with a viral load of 1,360 copies. Strict airborne, droplet, and contact precautions were implemented for infection control. The patient had also been started on a course of oral nirmatrelvir/ritonavir prior to transfer to our institution.

**Figure 1:**
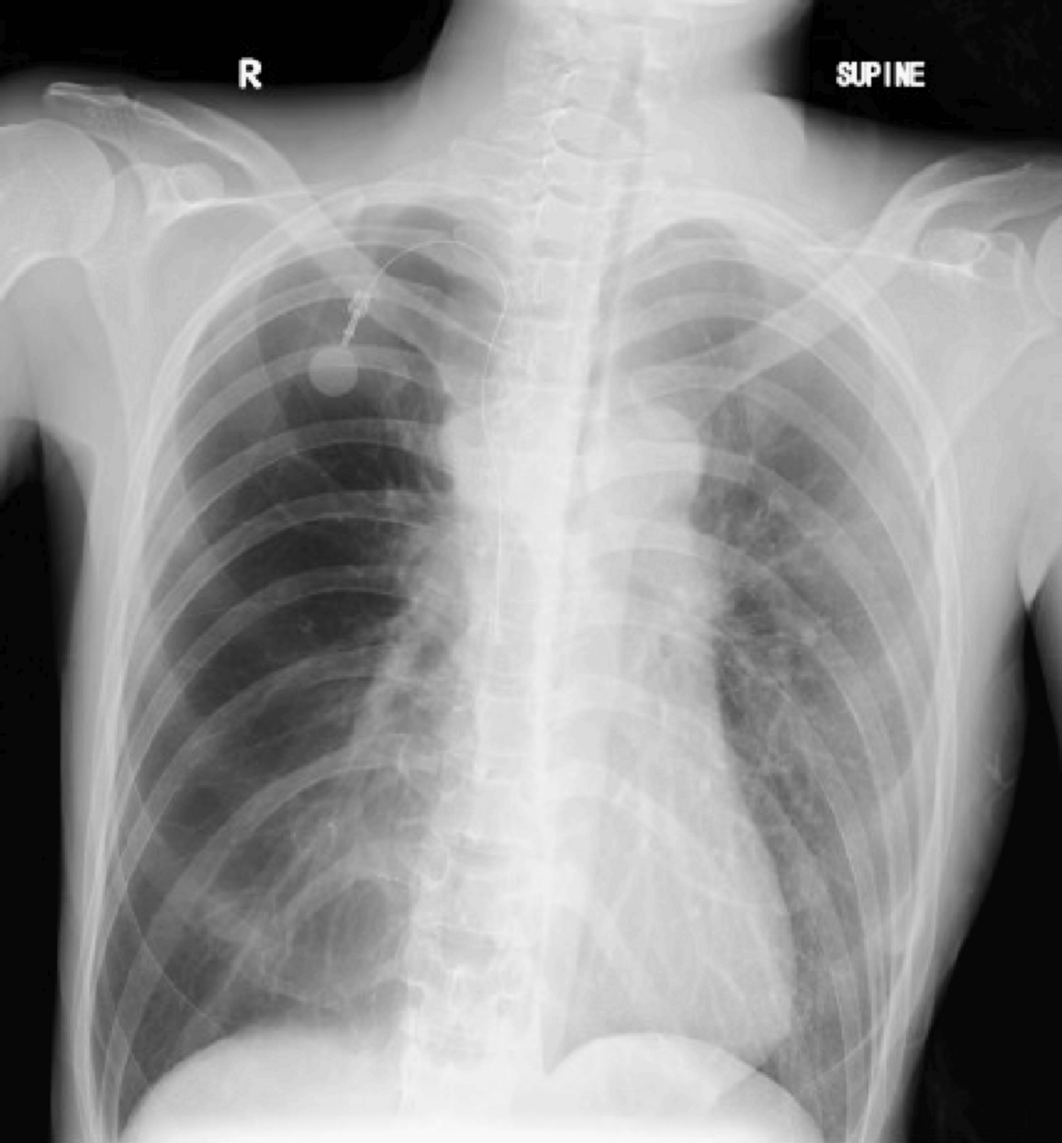
Preoperative chest radiograph The imaging was performed in the supine position. X-ray transparency is reduced in the left lung field, though this may be due to the patient's slightly oblique position

**Figure 2:**
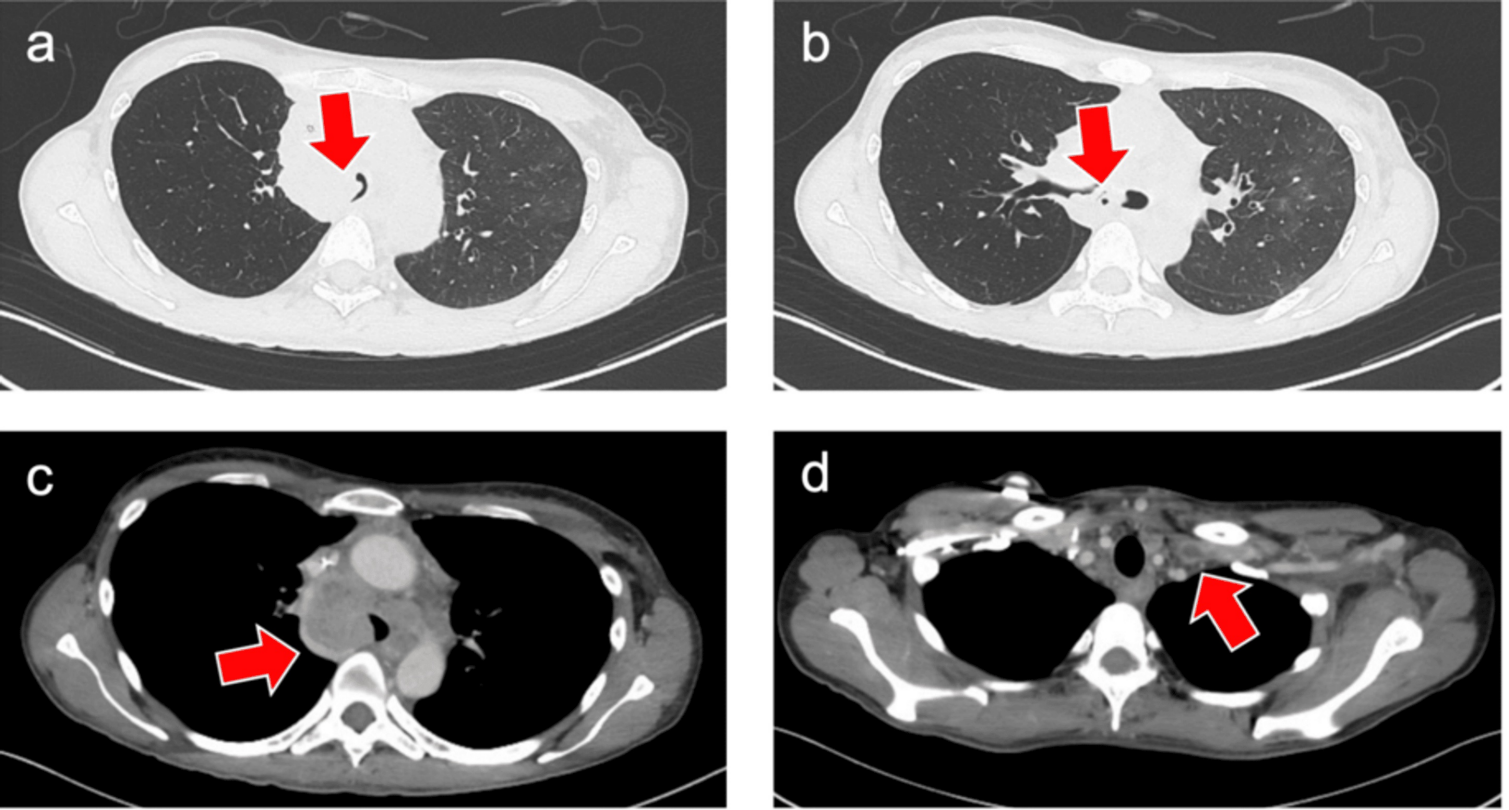
Contrast-enhanced chest computed tomography images (a) Enlarged mediastinal lymph node (red arrow) severely compressing the trachea from the right side. (b) Mediastinal lymph node (red arrow) extending into the right main bronchus. (c) Marked narrowing of the superior vena cava (red arrow). (d) Thrombus formation in the left subclavian vein (red arrow)

The preoperative chest radiograph showed a slightly oblique position and decreased X-ray transparency in the left lung field (Figure 1). Contrast-enhanced chest CT demonstrated numerous enlarged lymph nodes in the left cervical, bilateral supraclavicular, mediastinal, periaortic, and left iliac regions, consistent with suspected metastatic recurrence of small intestine carcinoma. Mediastinal lymphadenopathy caused severe tracheal compression, narrowing the lumen to approximately 2 mm (over a 90% reduction in cross-sectional area) at the level of the carina and extending into the right main bronchus (Figures 2a, 2b). Additionally, patchy ground-glass opacities were observed in the right upper, middle, and lower lobes, raising concern for COVID-19-associated pneumonia. The SVC was markedly narrowed (Figure 2c), with thrombus formation in the left brachiocephalic and subclavian veins (Figure 2d). Accordingly, the patient had been receiving a continuous infusion of heparin (10,000 units/day), which was discontinued eight hours before surgery.

The patient was diagnosed with severe tracheal stenosis secondary to mediastinal lymphadenopathy from recurrent small intestine carcinoma, SVC narrowing, thrombus formation in the left brachiocephalic and subclavian veins, and COVID-19-associated pneumonia. Although her initial oxygenation was preserved with low-flow supplemental oxygen, she exhibited worsening dyspnea, raising concern for impending airway compromise. Delaying intervention for severe tracheal stenosis was deemed unsafe due to the risk of acute respiratory failure, particularly in the setting of progressive tracheal compression and potential pneumonia exacerbation. Therefore, tracheal stent placement using rigid bronchoscopy was planned to alleviate airway obstruction.

**Figure 3:**
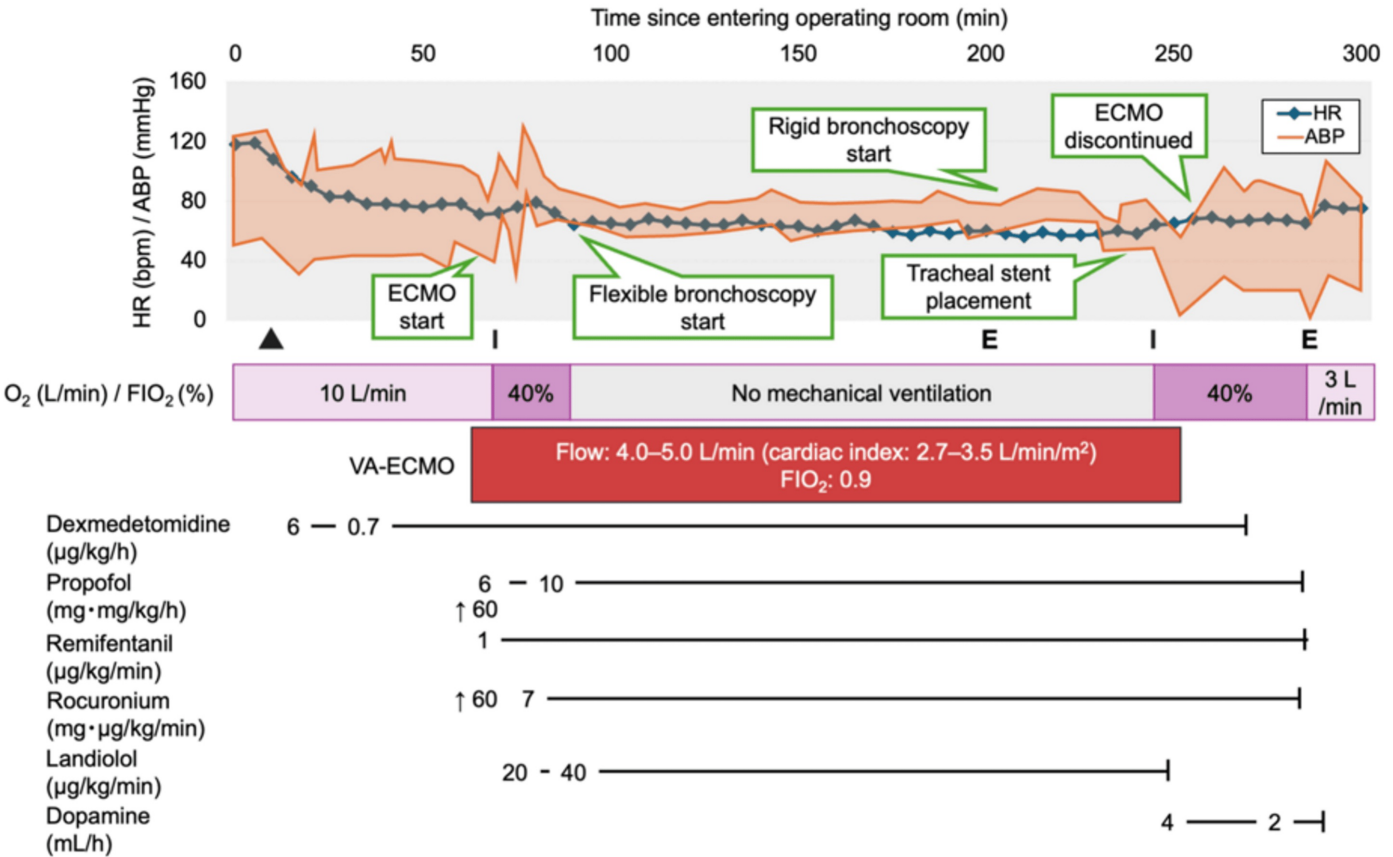
Intraoperative anesthetic record ▲: Ultrasound-guided peripheral nerve block (right quadratus lumborum block and right genitofemoral nerve block), I: tracheal intubation, E: extubation, ABP: arterial blood pressure, bpm: beats per minute, FIO_2_: fraction of inspired oxygen, HR: heart rate, VA-ECMO: venoarterial extracorporeal membrane oxygenation

Given the severe tracheal stenosis and worsening hypoxemia despite high-flow oxygen therapy, the cessation of spontaneous breathing during induction of general anesthesia posed an unacceptable risk of acute airway obstruction and asphyxia. Therefore, the primary strategy was to establish ECMO support before the induction of anesthesia to stabilize cardiopulmonary function. This approach also enabled apnea management during tracheal stent placement, eliminating the need for high-frequency jet ventilation and minimizing the risk of SARS-CoV-2 aerosolization. Regarding the ECMO configuration, although VV-ECMO is typically preferred for respiratory support, significant SVC narrowing and thrombus formation extending from the left brachiocephalic to the left subclavian vein precluded upper body venous access. Femorofemoral VV-ECMO was evaluated but avoided due to high recirculation risk. Therefore, femorofemoral VA-ECMO was selected, with a contingency plan to convert to veno-arterial-arterial (V-AA) ECMO by cannulating into the right axillary artery if differential hypoxia occurred. The procedure was performed in a negative-pressure operating room with full airborne, contact, and droplet precautions. All personnel wore caps, goggles, N95 respirators, face shields, long-sleeved gowns, and double gloves. The patient’s preexisting arterial and venous lines were utilized for intraoperative management: a 22-gauge catheter in the right radial artery and a 12-gauge quad-lumen central venous catheter in the left femoral vein. Monitoring included electrocardiography, noninvasive blood pressure, bilateral pulse oximetry, invasive arterial pressure via the radial artery line, regional cerebral oxygen saturation, and the bispectral index (BIS). Figure 3 presents the intraoperative anesthetic record. Under light sedation with continuous dexmedetomidine infusion, an ultrasound-guided right quadratus lumborum block and right genitofemoral nerve block were performed for analgesia at the cannulation sites. After confirming successful VA-ECMO establishment, general anesthesia was induced using propofol, remifentanil, and rocuronium. On arrival in the operating room, the baseline-activated clotting time (ACT) was 117 seconds. A bolus of 3,000 units of heparin was administered before cannulation, and the ACT was maintained at approximately 200 seconds.

**Figure 4:**
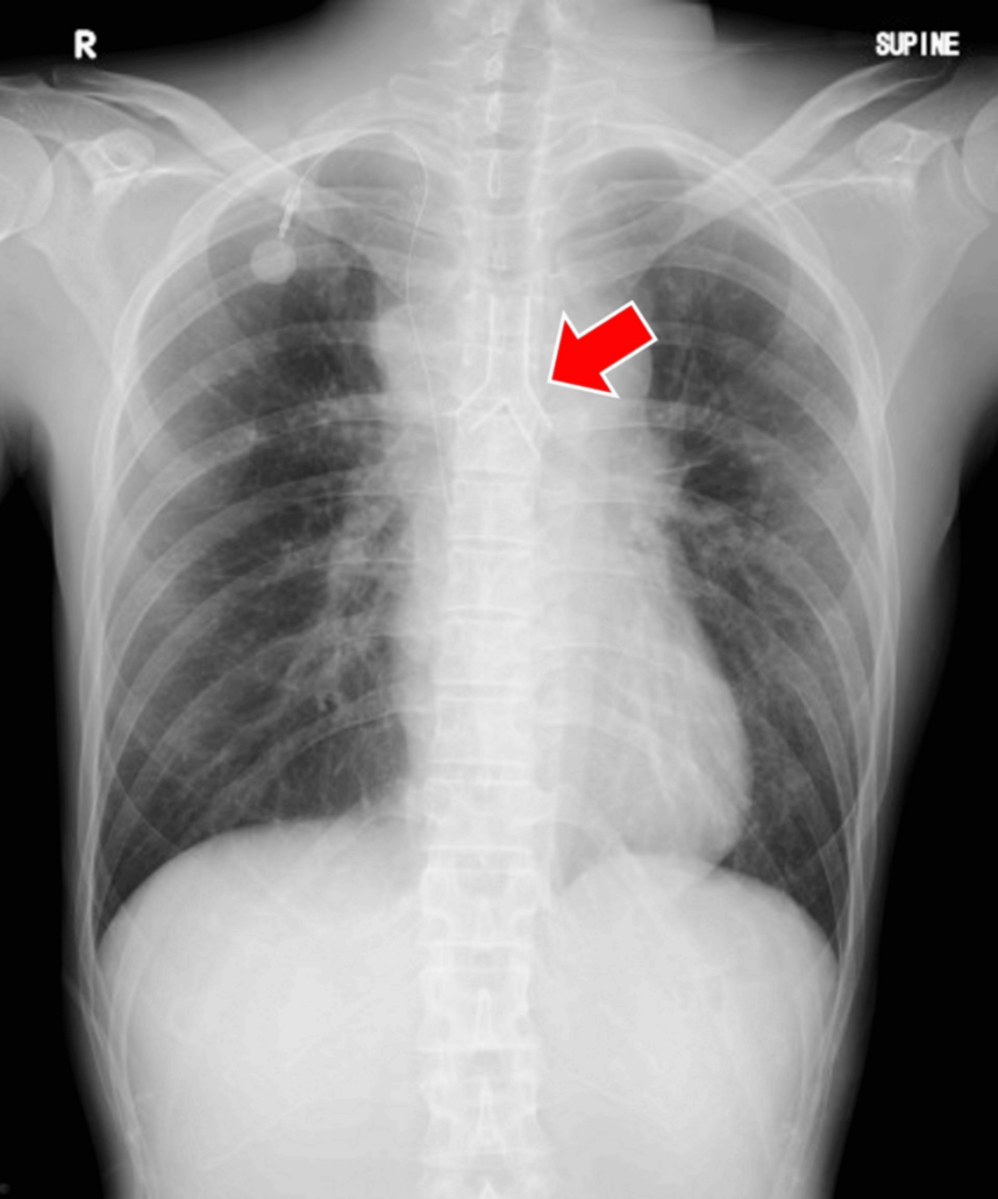
Chest radiograph after tracheal stent placement A Y-shaped silicone stent (red arrow) was positioned at the carina

Following tracheal intubation, the endotracheal tube was fixed at 20 cm from the incisors. X-ray fluoroscopy confirmed the tip lay 3 cm above the carina, 1 cm proximal to the tracheal stenosis. Before flexible bronchoscope insertion, the patient was managed on femorofemoral VA-ECMO under apneic conditions, and the following interventions were implemented to prevent differential hypoxia. First, VA-ECMO flow was maintained at 4.0-5.0 L/minute (cardiac index: 2.7-3.5 L/minute/m²), with a circuit fraction of inspired oxygen of 0.9. Second, deep anesthesia targeting a BIS of approximately 40 was achieved with continuous infusions of propofol at 6-10 mg/kg/hour and remifentanil at 1 μg/kg/minute. Third, native cardiac output was suppressed by a continuous infusion of landiolol, titrated up to 40 µg/kg/minute. During this period, the heart rate remained around 60 beats/minute, and the pulse pressure measured via the arterial line was approximately 10 mmHg. Fourth, in response to anemia (hemoglobin 7.2 g/dL), 560 mL of packed red blood cells were transfused to raise hemoglobin to approximately 9.0 g/dL. As a result of these interventions, oxygen saturation was maintained between 90% and 92% in the right hand and between 95% and 99% in the left hand. Regional cerebral oxygen saturation remained stable between 60% and 70%, consistent with preoperative baseline values. Once oxygenation was deemed sufficient, a flexible bronchoscope was inserted, revealing complete occlusion of the right main bronchus. Tumor ablation and balloon dilation were performed, followed by withdrawal of the bronchoscope and extubation. A rigid bronchoscope was subsequently introduced, and tracheal stent placement was initiated. A Y-shaped silicone stent (Dumon stent, Novatech S.A., France; outer diameter 14 mm, inner diameter 10 mm) was successfully placed under rigid bronchoscopy (Figure 4). During airway intervention under femorofemoral VA-ECMO support without mechanical ventilation, no ST-segment changes or arrhythmias were observed on electrocardiographic monitoring.

Then, the rigid bronchoscope was removed, the patient was reintubated, and mechanical ventilation resumed. Landiolol was discontinued, and VA-ECMO was weaned with dopamine support. General anesthetic agents were withdrawn, and the patient was extubated after confirming full recovery of consciousness and respiratory function. Postoperatively, the patient maintained adequate oxygenation on 3 L/minute oxygen via a face mask. No neurological complications were observed. Serum creatine kinase levels remained within the normal range throughout the perioperative course, indicating no myocardial injury. She was admitted to the intensive care unit (ICU), where her respiratory and hemodynamic status remained stable. She was discharged from the ICU on postoperative day 4 and returned to the referring hospital on day 8. She had been isolated due to a COVID-19 infection but was released on postoperative day 5. Notably, no healthcare professionals involved in the procedure developed COVID-19. The primary palliative goal of alleviating severe dyspnea was achieved, markedly improving her quality of life. She died of aortic perforation due to metastatic mediastinal lymphadenopathy on postoperative day 81.

## Discussion

It is important to emphasize that the decision to use VA-ECMO in this case was based on two concurrent life-threatening factors. First, the patient's severe tracheal stenosis (lumen narrowed to approximately 2 mm, over a 90% reduction in cross-sectional area) carried an extremely high risk of acute asphyxia during conventional induction of anesthesia and ventilation. Previous studies have indicated that active extracorporeal support is an effective safety strategy in situations of anticipated airway management difficulties or risk of asphyxia [10,11]. Therefore, establishing VA-ECMO before airway management procedures was the primary strategy to ensure airway safety. Second, the nonventilation strategy was adopted as the optimal approach for reducing SARS-CoV-2 aerosol exposure. Standard rigid bronchoscopy for stent placement typically requires high-frequency jet ventilation [2], which is considered an unacceptable high-risk aerosol-generating procedure in patients with active COVID-19 [12]. This infection-control decision was supported by evidence of a high transmission risk associated with high-frequency jet ventilation [13] and the possibility of breakthrough infections among vaccinated staff [14]. Although complex, this approach was judged the safest option to achieve the goal of alleviating respiratory distress in patients facing dual life-threatening challenges. Previous reports have described using VV-ECMO to facilitate apneic tracheal stent placement while minimizing aerosol generation [4]. In our case, severe narrowing of the SVC and thrombus formation in the left brachiocephalic and subclavian veins made upper body venous access infeasible. Additionally, the femorofemoral VV-ECMO strategy was avoided due to concerns regarding recirculation. VA-ECMO was selected as a feasible and safe alternative. Our experience demonstrates that, even with significant anatomical constraints, VA-ECMO can effectively support airway interventions without mechanical ventilation while maintaining adequate oxygenation and hemodynamic stability.

Differential hypoxia is a well-recognized complication during femorofemoral VA-ECMO, particularly when pulmonary function is impaired, and native cardiac output exceeds oxygenated ECMO flow. Strategies to prevent this include left ventricular venting or transitioning to hybrid ECMO configurations, such as V-AV or V-AA ECMO [7]. In our case, however, the patient was managed successfully without circuit modification by employing a multifaceted physiological approach. First, high VA-ECMO flow rates (4.0-5.0 L/minute) were maintained to maximize retrograde oxygen delivery from the femoral arterial cannula. Second, deep anesthesia was induced and maintained to reduce metabolic demand, with a target BIS of approximately 40. Third, native cardiac output was suppressed by a continuous infusion of landiolol, an ultrashort-acting β₁-selective blocker, titrated up to 40 µg/kg/minute. The use of intravenous β-blockade to reduce left ventricular output and mitigate differential hypoxia during femorofemoral VA-ECMO has been described previously [15]. This pharmacologic strategy limits the competitive ejection of poorly oxygenated blood from the left ventricle, enhancing the effectiveness of ECMO-derived retrograde oxygenation. Fourth, anemia was corrected by transfusing packed red blood cells (560 mL) to improve oxygen-carrying capacity and systemic delivery. Using these combined interventions, right-hand oxygen saturation was maintained between 90% and 92%, and regional cerebral oxygen saturation remained in the preoperative range. It is important to recognize, however, that coronary circulation may still be compromised even when upper body oxygenation appears adequate. As highlighted in a recent report of cardiac Harlequin syndrome [16], if the mixing zone of ECMO and native blood lies proximal to the coronary ostia, desaturated blood may perfuse the myocardium, potentially leading to ischemia, ST-segment elevation, and ventricular arrhythmias. No such adverse cardiac events occurred in our case; however, this underscores the need for vigilant electrocardiographic and hemodynamic monitoring during apneic femorofemoral VA-ECMO support.

This is one of the few reports describing the successful prevention of differential hypoxia during apneic femorofemoral VA-ECMO airway intervention without requiring arterial repositioning or additional cannulation strategies. To our knowledge, this is the first reported case of tracheal stent placement under femorofemoral VA-ECMO support without mechanical ventilation in a patient with active SARS-CoV-2 infection. This case underscores the clinical value of adapting airway management strategies to both patient-specific anatomic challenges and infection control needs in the COVID-19 era. Tracheal stenting without mechanical ventilation reduced the risk of aerosol generation, protecting healthcare professionals without compromising procedural success. Importantly, this was accomplished in a high-risk patient with central airway obstruction and COVID-19 pneumonia, conditions that each independently complicate anesthetic management. The use of a negative-pressure operating room, enhanced personal protective equipment, and specialized monitoring protocols contributed to procedural safety. Notably, no members of the surgical or anesthetic team developed COVID-19 following the procedure, supporting the effectiveness of our infection control strategy. Furthermore, this management strategy may be applicable not only to COVID-19 but also to any future pandemic involving novel, highly transmissible airborne pathogens, providing a robust framework for similar high-risk airway procedures.

In this case, the tracheal stent was placed with palliative intent to relieve her debilitating dyspnea and avert imminent asphyxiation. This objective was achieved, leading to a significant improvement in her quality of life. The patient's postoperative survival was 81 days. This duration is consistent with the reported median survival of three to four months for patients undergoing palliative airway stenting for malignant central airway obstruction [17,18]. This suggests that our intervention allowed the patient to receive the full anticipated palliative benefit from securing the airway before her underlying malignancy progressed to its terminal stage.

## Conclusions

In summary, this case demonstrates that tracheal stent placement under apneic femorofemoral VA-ECMO support is a safe option for patients with critical airway obstruction complicated by COVID-19, particularly when conventional VV-ECMO is not feasible. Proactive ECMO provided essential cardiorespiratory support to avert asphyxiation during anesthesia induction, while simultaneously enabling an apneic technique that minimized viral aerosolization. Combining deep sedation, cardiac output suppression, anemia correction, and high-flow ECMO prevented differential hypoxia during femorofemoral VA-ECMO support. However, this was a single case report; therefore, the generalizability of this management strategy requires validation through further case accumulation.
